# Joseph P. Root

**Published:** 1912-10-15

**Authors:** 


### OBITUARY.

Joseph P. Root, Editor of Western Dental Journal and friend of every one who knew him died September 4, 1912. Many dentists will be shocked to learn of the untimely death of one of their best known and loved professional brothers. We do not at this writing know the particulars, except that his death was caused by an internal injury from playing golf. The following tribute from the pen of Dr. Hungerford will find a hearty approval of all who ever came in contact with him.

J. P. Root, "formerly of Kansas"—all is said. It was a slogan to conjure with. Wherever dentists were congregated together there was Joe in the midst of them; were reforms to be instituted, abuses to be corrected, Joe's voice was foremost in the debate, ever on the side of right. North, East, South and West, his friends were legion, and they who knew him best loved him most. Time, the All-Healer, alone can fill the wound untimely death has made.

Joe was in love with life and life was joy; his presence was a disperser of gloom, and many a weary heart to-day has cause to be thankful that Joseph was his friend. Eye to eye and shoulder to shoulder, Joe fought in the ranks of those who are winning humanity's battles.

In the processes of the Great Law, Death has its part; all things are cyclic, and the recurring periods of rest, which we call death, are as natural as the cycled sleep of the flowers. Not forever can the soul live and grow and experience in one poor body, and so it slips away, again and yet again laying aside that body as one lays aside a garment that is old, each time does the soul rise into larger view. Witnessing the present life as one of many lives, what joy is born of this larger view. All that is now unrealized will be realized in lives that are to come—old ties to be recemented, old comrades welcomed and embraced, and though the heart must hunger for a time, in spite of the soul's assurance, the inner voice will speak of harmonies sublime. A soul has crossed the portal and stepped out into its real life, the life that is unweighted by disappointment or despair—in which all hopes are realized.

The only heaven we can enjoy is the one we have prepared for ourselves. To the man whose thoughts were less of self than how he could assuage his brother's grief, death comes as a white-robed messenger of peace, with cheerful mien and tender hands, to lead him to his well-earned repose.

In affectionate memory of a friend and comrade.—C. L. H.

